# A Meta-Analysis of Antiviral Therapy for Hepatitis B Virus-Associated Membranous Nephropathy

**DOI:** 10.1371/journal.pone.0160437

**Published:** 2016-09-06

**Authors:** Yue Yang, Ye-ping Ma, Da-peng Chen, Li Zhuo, Wen-ge Li

**Affiliations:** Department of nephrology, China-Japan friendship hospital, Beijing, PR China; University of the Witwatersrand, SOUTH AFRICA

## Abstract

Hepatitis B virus-associated membranous nephropathy (HBV-MN) is the most common renal extra-hepatic manifestation in patients with chronic HBV infection. In September 2015, we searched the MEDLINE, EMBASE, and CENTRAL databases, and the reference lists of retrieved articles, to identify relevant studies. Descriptions of antiviral drugs used to treat HBV-MN were included in our review. Two authors independently screened all relevant articles, extracted data, and assessed the risk of bias. Nine hundred and fifty-four papers have been considered after electronic and manual searching, only five relevant studies were identified. Complete remission (OR = 26.87, 95% CI: 8.06 to 89.52), total remission (OR = 10.31, 95% CI: 3.59 to 29.63) of proteinuria and HBeAg clearance (OR = 20.91, 95% CI: 6.90 to 63.39) increased significantly after antiviral therapy. No significant differences were seen between interferon and nucleoside analog treatments. Our study found that antiviral therapy was an effective treatment in HBV-MN patients; interferon and nucleoside analogs were equally effective at causing proteinuria remission and HBeAg clearance.

## Introduction

Hepatitis B virus (HBV) is globally distributed; approximately one-third of the world’s population exhibits serological evidence of past or present HBV infection[1]. About 240 million subjects are chronic HBV surface antigen (HBsAg) carriers, rendering HBV one of the most common human pathogens[2]. The prevalence of HBV infection varies significantly in different regions of the world[3], infection is most prevalent in sub-Saharan Africa and Asia[4]. However, HBV is also found in migrant populations and second-generation immigrants to developed countries.

Many types of extra-hepatic disease manifestations have been observed in patients with acute or chronic hepatitis caused by HBV. In 1971, Combes et al.[5] described a 53-year-old male with membranous nephropathy (MN) attributable to glomerular deposition of Australian-antigen-containing immune complexes, this was the first report of hepatitis B virus- associated glomerulonephritis (HBV-GN).

HBV-MN is the most common pathological category of HBV-GN, and proteinuria is the most common clinical manifestation of HBV-MN[4, 6]. Persistent severe proteinuria triggers renal failure rapidly. Routinely, antivirals are used to treat HBV, and both interferon(IFN) and nucleoside analogs (NAs) have been approved by the Food and Drug Administration for treatment of adults with HBV[7]. The objective of this study is to determine the benefits of antiviral drugs with HBV-MN patients, and compare the effect between IFN and NAs.

## Methods

We performed our meta-analysis as suggested by the Cochrane guidelines[8] and adhered to the relevant criteria of PRISMA (Preferred Reporting Items for Systematic Reviews and Meta-Analyses)[9], check list of PRISMA was shown in S1 Table. Protocol and registration information were available on http://www.crd.york.ac.uk/PROSPERO/ (CRD42015026939).

### 1. Search strategy

We searched the MEDLINE, EMBASE, and CENTRAL databases for relevant works in the English language, the cutoff date for database inclusion was end-September 2015. To ensure the comprehensive, accurate retrieval of studies, a comprehensive search strategy was established (S2 Table). We also manually checked the reference lists of nephrology textbooks, review articles, all retrieved studies, and reports of academic congresses.

### 2. Selection criteria

We included data on all HBV-infected patients with HBV-MN irrespective of age or sex. Studies meet the following criteria were included in our research: (1) patients with HBV-MN, HBV-MN was diagnosed when immunofluorescent staining of renal biopsy tissue revealed diffuse granular capillary membranous deposits containing at least one of HBsAg, HBcAg, and/or HBeAg; (2) Long term administration of antiviral therapy (IFN or NAs), with a period of follow up ≥ 6 months. Clinical trials meeting the following criteria were excluded: (1) studies include various pathological types of HBV-GN; (2) self control studies or case reports; (3) HBV infection but not HBV-MN; (4) not reported as an original article.

### 3. Selection of studies and data extraction

The search identified all eligible studies. The titles and abstracts were screened by two investigators (Y. Yang and Y. Ma) who independently assessed the abstracts, and if necessary the full texts, to determine whether the studies satisfied the inclusion criteria. If the views of these two investigators differed, a third investigator (L. Zhuo) read the study in question, all three investigators then engaged in discussion. The study was included only if consensus was achieved.

Data extraction was performed independently by Yang and Ma, using standard forms. The following variables were extracted: (1) complete remission (CR) and partial remission (PR) of proteinuria; (2) HBeAg clearance.

### 4. Risk of bias assessment

Two investigators (Yang and Ma) independently assessed risk of bias using the Cochrane risk-of-bias tool[8] with randomized control trial (RCT) and the Newcastle-Ottawa scales[10] with observational cohort study. For RCT, we reviewed each trial and scored it as high, low, or unclear risk of bias to the following criteria: random sequence generation, allocation concealment, blinding of participants and personnel to the study protocol, blinding of outcome assessment, incomplete outcome data, selective reporting, and other bias. For observational cohort studies, in brief, a maximum of 9 stars was assigned to each study, 4 for selection (representativeness of the exposed cohort, selection of the non-exposed cohort, ascertainment of exposure, demonstration that outcome of interest was not present at start of study), 2 for comparability (comparability of cohorts on the basis of the design or analysis), and 3 for outcomes (assessment of outcome, was follow-up long enough for outcomes to occur, adequacy of follow up of cohorts), we considered a study awarded six or more as a high-quality study.

### 5. Statistical analysis

We calculated odds ratio (OR) with 95% CIs for CR, PR of proteinuria and clearance of HBeAg. The fixed effect model was used for the meta-analysis of each indicator. Subgroup analyses were performed between IFN and NAs. The heterogeneity test was performed using the chi-square test and the *I*² statistic, *I*^2^ > 50% indicated significant heterogeneity[11]. *P* < 0.05 indicated a statistically significant difference. Publication bias was assessed by visually inspecting a funnel plot. All statistical analyses were performed using RevMan 5.3 (Nordic Cochrane Centre).

## Results

### 1. Basic information regarding the enrolled studies

**Fig 1:**
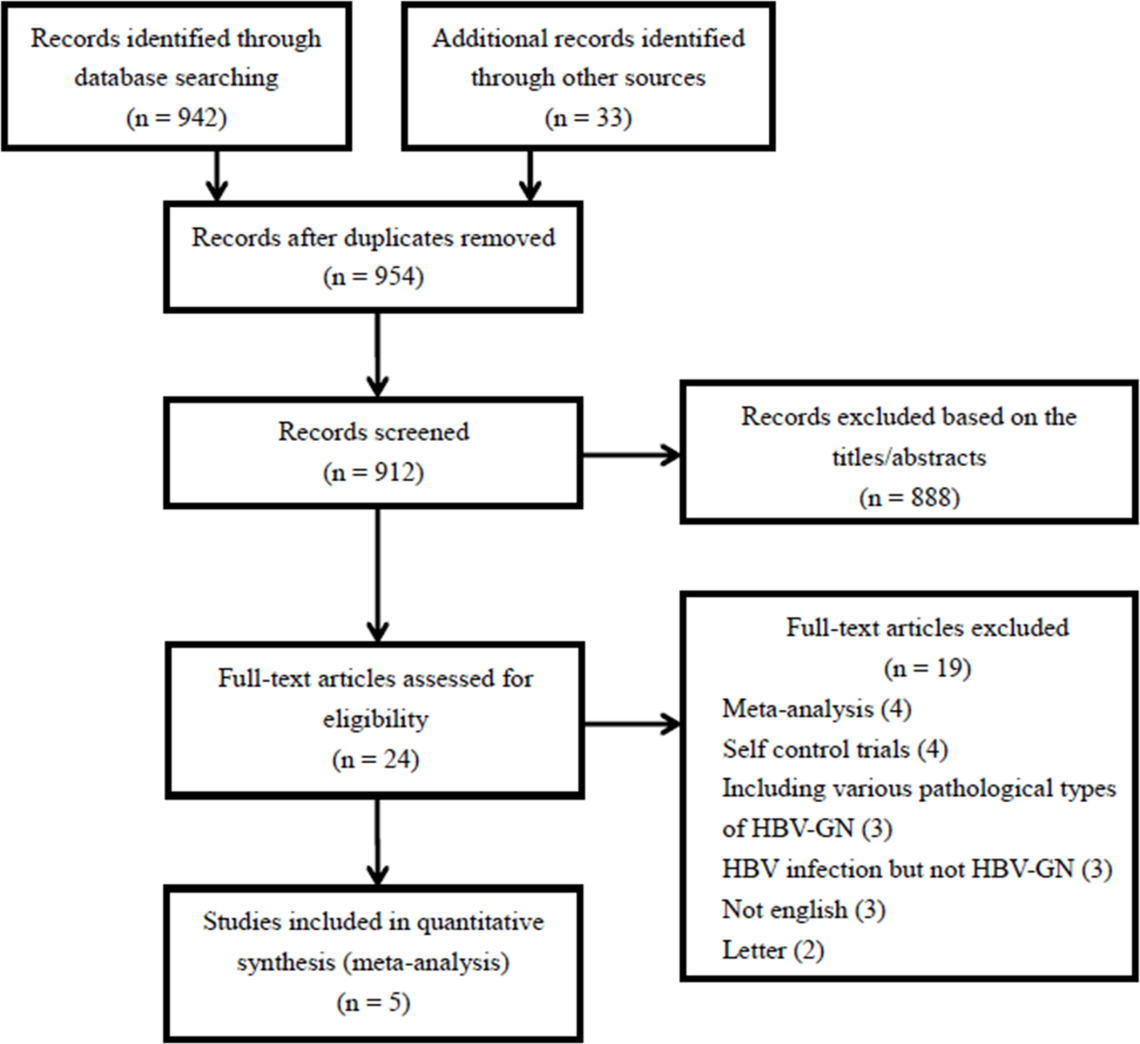
Flow diagram of study identification, inclusion and exclusion.

We retrieved 942 records electronically, and a further 33 studies by exploring additional sources. The potentially relevant studies numbered 954 after duplicates were removed. Studies that met the following criteria were included after preliminary screening: (1) articles with HBV and glomerular nephritis; (2) treated with antiviral drug or other therapy. Then, we shortlisted 24 papers for detailed evaluation. Ultimately, only five studies were found to be eligible. Details of full text screening and the study selection process are illustrated in Fig 1, detailed information regarding the included studies is provided in Table 1.

**Table 1:** Characteristics of the included studies.

Author	Year	Region	Age (y)	Treatment	N	Duration	Follow-up	Design	Dropout (n)
Bhimma [12]	2002	Durban, South Africa	8.7	IFN	19	16 weeks	40 weeks	Co	5
			9.2	Control^a^	20				
Lai [13]	1991	Hong Kong, China	16–47	IFN	5	12 weeks	25–108 months	Co	0
			15–53	No treatment	11		24–108 months		
Lin[14]	1995	Taiwan, China	6.2±2.4	IFN	20	1 year	24 months	RCT	0
			6.8±2.1	Control^a^	20				
Sun [15]	2012	Seoul, Korea	19–64	NAs	6	NA	8–60 months	Co	0
				Control^a^	4				
Tang [16]	2005	Hong Kong, China	48.3±12.8	NAs	10	NA	49.2±16.5 months	Co	0
			43.1±22.8	Control^a^	12		188±88 months		

Data are expressed as mean or mean ± SD. IFN, interferon; NAs, nucleoside analogs; NA, not available; Co, cohort study; RCT, random control trial.

^a^ In order to control the oedema, hypertension and other symptoms, controls received the same treatment as study patients except antiviral drug.

### 2. Risk of bias

**Fig 2:**
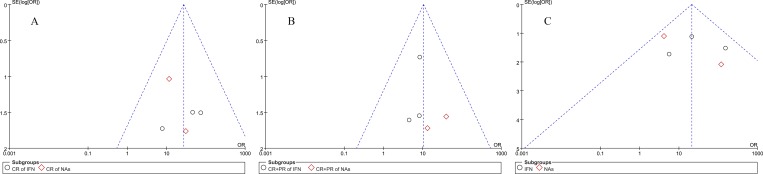
Publication bias analysis with funnel plots. A: Complete remission with IFN and NAs treatment; B: Total remission (complete remission and partial remission) with IFN and NAs treatment; C: clearance of HBeAg after IFN and NAs treatment.

The only RCT[14] in our analysis is low risk with random sequence generation, blinding of participants and personnel, blinding of outcome assessment and incomplete outcome data, high risk with allocation concealment, and unclear risk with selective reporting. Biases of observational cohort studies with Newcastle-Ottawa scale is shown in Table 2. All studies had 6 or more stars and regarded as high quality. Funnel plots in Fig 2 shows a symmetric inverse funnel distribution indicating no significant publication biases were detected in the meta-analyses of this investigation.

**Table 2:** Newcastle-Ottawa scale of observational studies.

Study, Year	Selection (up to 4)	Comparability (up to 2)	Outcome (up to 3)
Bhimma [12], 2002	3	1	2
Lai[13], 1991	2	1	3
Sun[15], 2012	3	1	3
Tang [16], 2005	3	1	3

### 3. Outcomes

#### 3.1 Complete and partial remission following antiviral therapy

**Fig 3:**
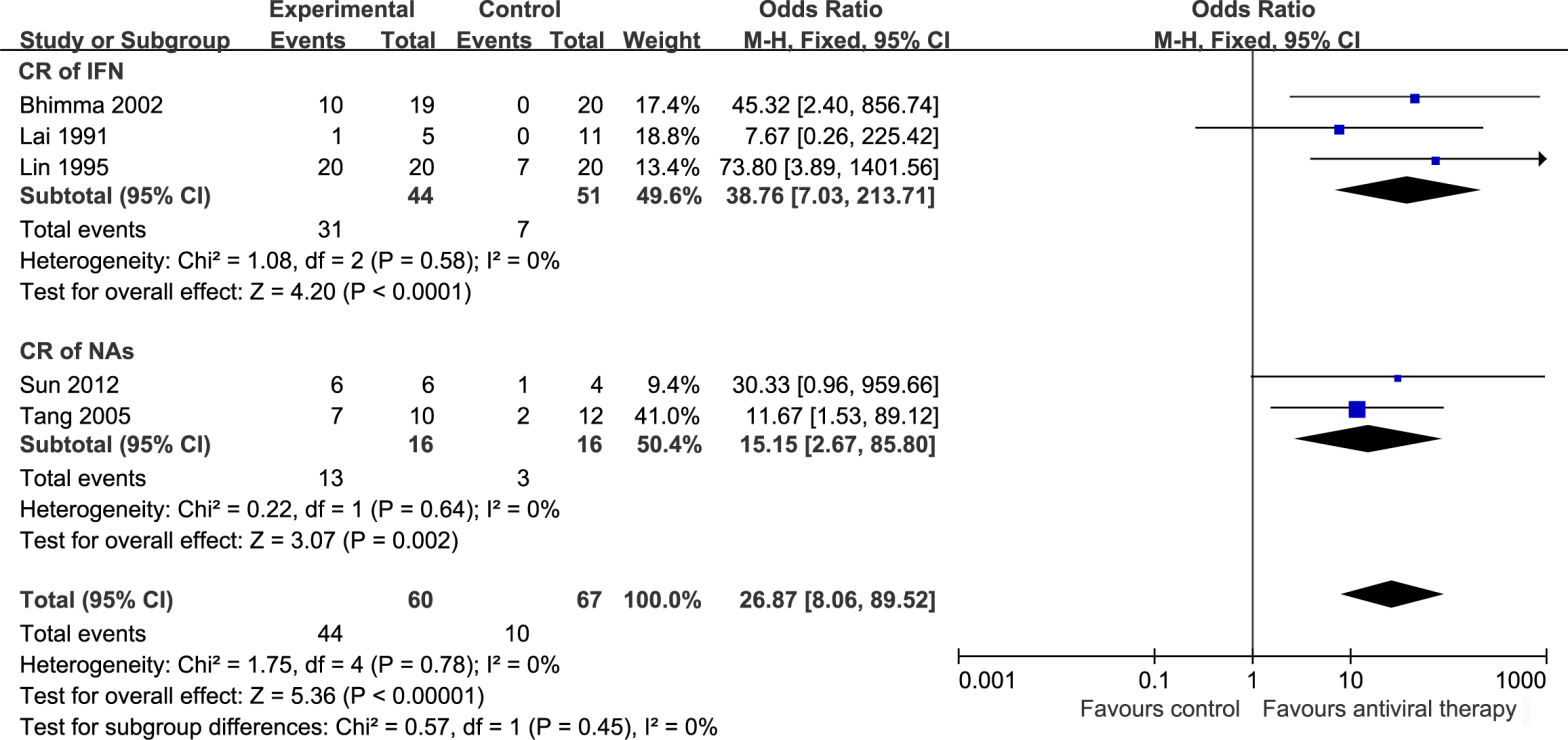
CR of antiviral therapy on HBV-MN. OR: Odds ratio, CR: Complete remission, IFN: interferon, NAs: nucleoside analogs.

**Fig 4:**
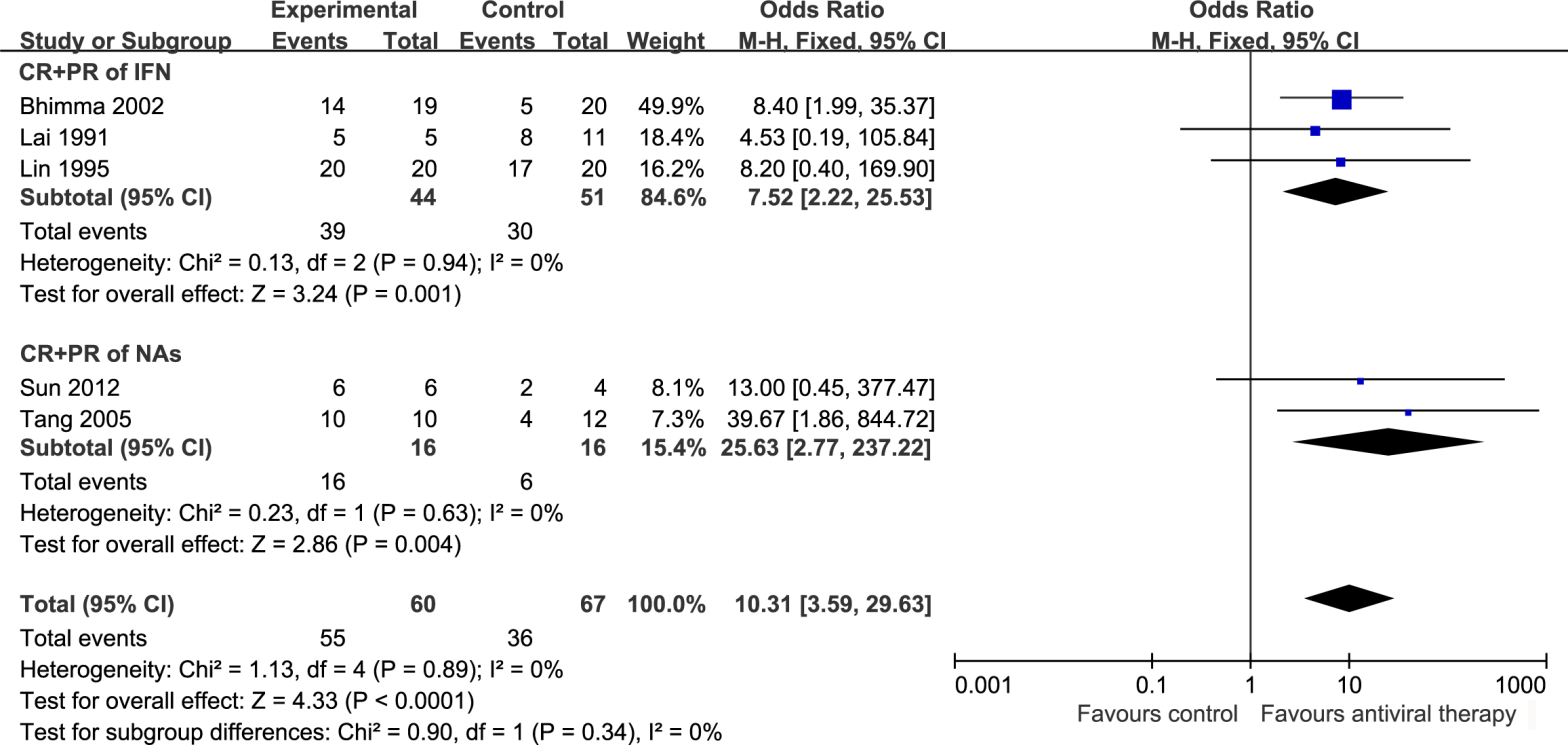
CR and PR of antiviral therapy on HBV-MN. OR: Odds ratio. CR: Complete remission. PR: Partial remission, IFN: interferon, NAs: nucleoside analogs.

The efficacy of antiviral therapy on proteinuria with HBV-MN patients was assessed using 5 trials, including 1 RCT[14] and 4 observational cohort studies[12, 13, 15, 16]. These studies included a total of 127 cases. To compare the effect of IFN and NAs, we set two subgroups. We evaluated the CR rate after treatment, heterogeneity using the *I*^2^ statistic was 0%, *P* = 0.78, the test for overall effect was *P* < 0.00001 with a fixed effect model. After antiviral therapy proteinuria was significantly decreased (OR = 26.87, 95% CI: 8.06 to 89.52) but there was not significant difference between IFN (OR = 38.76, 95% CI: 7.03 to 213.71) and NAs (OR = 15.15, 95% CI: 2.67 to 85.80) treatments (Fig 3). Result of total remission which included CR and PR was similar to CR (Fig 4).

After antiviral therapy proteinuria was significantly decreased (OR = 26.87, 95% CI: 8.06 to 89.52) but there was not significant difference between IFN (OR = 38.76, 95% CI: 7.03 to 213.71) and NAs (OR = 15.15, 95% CI: 2.67 to 85.80) treatments (Fig 3).

#### 3.2 Clearance of HBeAg in antiviral therapy

**Fig 5:**
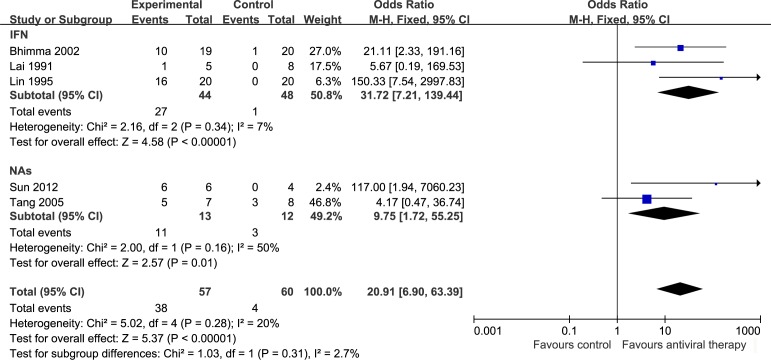
HBeAg clearance of antiviral therapy on HBV-MN. OR: Odds ratio, IFN: interferon, NAs: nucleoside analogs.

The efficacy of antiviral therapy on HBeAg clearance with HBV-MN patients was assessed using 5 trials[12–16], including a total of 127 cases. We evaluated the HBeAg clearance after antiviral therapy, heterogeneity using the *I*^2^ statistic was 20%, *P* = 0.28, the test for overall effect was *P* < 0.00001 with a fixed effect model. HBeAg was significant decrease after antiviral therapy (OR = 20.91, 95% CI: 6.90 to 63.39), but no significant difference between IFN (OR = 31.72, 95% CI: 7.21 to 139.44) and NAs (OR = 9.75, 95% CI: 1.72 to 55.25) treatments (Fig 5) were noted.

### 1. Tolerability and safety

Patients treated with IFN commonly developed a flu-like illness and fever, and psychiatric problems were also observed in some cases. None of the included studies reported the occurrence of any serious adverse reactions after treatment with NAs.

## Discussion

The meta-analysis results revealed that antiviral therapy in HBV-MN patients is effective at causing proteinuria remission and HBeAg clearance, while there was no significant difference between interferon (IFN) and nucleoside analogs (NAs).

Several meta-analyses[17–19] have focused on the effects of different therapies in HBV-GN patients, which include MN, membranoproliferative glomerulonephritis and mesangial proliferative glomerulonephritis. Therefore, the heterogeneity of the response to antiviral therapy for different types of glomerulonephritis makes it difficult to establish conclusions regarding the recommendation of antiviral drugs in this population. In our meta-analysis, all but two of the patients (125/127, 98.4%) had MN. In Bhimma’s study[12], one patient had MPGN and the other was not determined. Ours is the first meta-analysis of HBV-MN that includes treatment with IFN and NAs.

In Yi[20], the patients were subdivided according to patient age, and the incidence of proteinuria increased significantly in both adults and children. According to the KDIGO clinical practice guidelines for glomerulonephritis[21], patients with HBV infection and glomerulonephritis should receive treatment with IFN or NAs, although no further comparison was made. Comparing IFN versus NAs, we found that the effects of these two types of drug were quite similar. Note that our analysis included only one RCT, so the evidence is insufficient and further trials are required.

During the course of HBV infection, the loss of serum HBeAg and development of antiHBe antibodies (HBeAg seroconversion) mark a transition from the immune-active phase of the disease to the inactive carrier state[22]. IFN is an antiviral drug commonly used to treat HBV, due to its dual effects of immunoregulation and viral suppression[23]. The dosage and treatment duration also have important effects on the clinical outcome.

Compared with IFN, NAs were easy to take and used widely. Lamivudine is the most popular NA, one potential limitation of prolonged lamivudine treatment is the emergence of drug-resistant HBV strains; the frequency of this increases with time[24], although patients who had lamivudine-resistant strains with mutations at the YMDD motif of the DNA polymerase also developed complete remission of the proteinuria after another NA was added[15].

Steroids or even immunosuppressive agents were also used in some HBV-MN cases, but most of these were case reports[25] or self-control studies[19]. Steroids and immunosuppressive agents may trigger transient viral replication associated with increased serum levels of HBeAb and HBV-DNA[26] and does not reduce the proteinuria or promote HBeAg clearance [18].

The side effects of IFN-α therapy include a flu-like syndrome, fever, and fatigue. Most side effects are well tolerated, but there are some serious adverse effects, such as psychiatric symptoms. When patients develop serious adverse effects, the dosage of IFN should be reduced immediately. Lamivudine is well tolerated; however, the emergence of drug-resistant viral strains is of concern.

## Conclusions

In summary, our meta-analysis showed that antiviral therapy is an effective regimen for patients with HBV-MN. IFN and NAs have different characteristics, but both were effective in terms of proteinuria remission and HBeAg clearance. Future, high-quality, large-scale RCTs should provide more reliable results for evidence-based medicine and the clinical drug treatment of HBV-MN patients.

## Supporting Information

S1 Table PRISMA 2009 checklist. (DOC)

S2 Table Search strategies for databases. (DOC)
