# Supplementary material for: A Meta-Analysis of Antiviral Therapy for Hepatitis B Virus-Associated Membranous Nephropathy
Source: PLoS One. 2016 Sep 6;11(9):e0160437. doi: 10.1371/journal.pone.0160437 (PMC5012684; doi:10.1371/journal.pone.0160437)
Supplement: S2 Table — (DOC) [file pone.0160437.s002.doc]

**Supporting table 2. S**earch strategies for databases

| MEDLINE | EMBASE | CENTRAL |
| --- | --- | --- |
| 1. “[Hepatitis B virus](http://www-ncbi-nlm-nih-gov.ezproxy.med.nyu.edu/pubmed/24701032)”[MeSH] 2. “[Hepatitis B](http://www-ncbi-nlm-nih-gov.ezproxy.med.nyu.edu/pubmed/24701032), chronic” [MeSH] 3. “[Glomerulonephritis](http://www-ncbi-nlm-nih-gov.ezproxy.med.nyu.edu/pubmed/24701032)”[MeSH] 4. “hepa­titis B virus associated glomerulonephritis” OR HBV-GN 5. ((#1 OR #2) AND #3) OR #4 | 1. ‘[Hepatitis B virus](http://www-ncbi-nlm-nih-gov.ezproxy.med.nyu.edu/pubmed/24701032)’/exp 2. ‘[Hepatitis B](http://www-ncbi-nlm-nih-gov.ezproxy.med.nyu.edu/pubmed/24701032), chronic’/exp 3. ‘[Glomerulonephritis](http://www-ncbi-nlm-nih-gov.ezproxy.med.nyu.edu/pubmed/24701032)’/exp 4. ‘hepa­titis B virus associated glomerulonephritis’ OR HBV-GN 5. ((#1 OR #2) AND #3) OR #4 | 1. MeSH descriptor [Hepatitis B virus](http://www-ncbi-nlm-nih-gov.ezproxy.med.nyu.edu/pubmed/24701032) explode all trees 2. MeSH descriptor [Hepatitis B](http://www-ncbi-nlm-nih-gov.ezproxy.med.nyu.edu/pubmed/24701032), chronic, this term only 3. MeSH descriptor [Glomerulonephritis](http://www-ncbi-nlm-nih-gov.ezproxy.med.nyu.edu/pubmed/24701032) explode all trees 4. (hepa­titis B virus associated glomerulonephritis):ti,ab,kw in Clinical Trials 5. (HBV-GN):ti,ab,kw in Clinical Trials 6. ((#1 OR #2) AND #3) OR #4 OR #5 |
